# Supplementary material for: Deletion of wfs1 Impairs Oligodendrocyte Precursor Cells Dorsal Distribution and Myelination Through the wfs1-hmgcs1 Axis in Zebrafish
Source: Biology (Basel). 2026 Aug 16;15(16):1402. doi: 10.3390/biology15161402 (PMC13510151; doi:10.3390/biology15161402)
Supplement: Supplementary file 1 [file biology-15-01402-s001.zip › File S1-Transcriptome sequencing data and differentially expressed genes (DEGs) in wfs1-- zebrafish at 5 dpf/2.KEGG/wfs1KOvsWT/GSEA/WT/BIOSYNTHESIS_OF_AMINO_ACIDS/BIOSYNTHESIS_OF_AMINO_ACIDS(DRE01230).html]

Details for gene set BIOSYNTHESIS\_OF\_AMINO\_ACIDS(DRE01230)[GSEA]

|  || Dataset | deg.sample |
| Phenotype | sample.cls |
| Upregulated in class | WT |
| GeneSet | BIOSYNTHESIS\_OF\_AMINO\_ACIDS(DRE01230) |
| Enrichment Score (ES) | -0.28491753 |
| Normalized Enrichment Score (NES) | -1.2748314 |
| Nominal p-value | 0.12576064 |
| FDR q-value | 0.49596053 |
| FWER p-Value | 0.987 |
Table: GSEA Results Summary

  

Fig 1: Enrichment plot: BIOSYNTHESIS\_OF\_AMINO\_ACIDS(DRE01230)      
 Profile of the Running ES Score & Positions of GeneSet Members on the Rank Ordered List

  

| PROBE | DESCRIPTION (from dataset) | GENE SYMBOL | GENE\_TITLE | RANK IN GENE LIST | RANK METRIC SCORE | RUNNING ES | CORE ENRICHMENT || 1 | 560753\_1 | tpi1b |  |  | 49 | 4.890 | 0.0514 | No |
| 2 | 560827\_1 | prps1b |  |  | 229 | 4.021 | 0.0892 | No |
| 3 | 334195\_1 | mat1a |  |  | 452 | 3.119 | 0.1160 | No |
| 4 | 561416\_1 | pfkpb |  |  | 497 | 2.998 | 0.1470 | No |
| 5 | 445818 | cthl |  |  | 799 | 2.387 | 0.1634 | No |
| 6 | 100006589\_1 | idh1 |  |  | 876 | 2.275 | 0.1856 | No |
| 7 | 393799 | pycr1a |  |  | 890 | 2.250 | 0.2095 | No |
| 8 | 550579\_1 | idh3g |  |  | 1275 | 1.830 | 0.2173 | No |
| 9 | 58068 | pcxb |  |  | 1914 | 1.406 | 0.2124 | No |
| 10 | 406688\_1 | got2a |  |  | 2167 | 1.300 | 0.2185 | No |
| 11 | 563642 | sdsl |  |  | 2618 | 1.139 | 0.2167 | No |
| 12 | 407673\_1 | rpia |  |  | 2807 | 1.082 | 0.2225 | No |
| 13 | 114551 | pklr |  |  | 2861 | 1.068 | 0.2324 | No |
| 14 | 101886255 | LOC101886255 |  |  | 2978 | 1.042 | 0.2400 | No |
| 15 | 322670 | aco2 |  |  | 3094 | 1.015 | 0.2474 | No |
| 16 | 550524 | cbsa |  |  | 3402 | 0.952 | 0.2480 | No |
| 17 | 406696 | pgk1 |  |  | 3987 | 0.833 | 0.2386 | No |
| 18 | 560682 | pycr3 |  |  | 4000 | 0.831 | 0.2473 | No |
| 19 | 791612 | mat2ab |  |  | 4341 | 0.771 | 0.2449 | No |
| 20 | 560944 | pfkpa |  |  | 4480 | 0.748 | 0.2486 | No |
| 21 | 568001\_3 | pfkmb |  |  | 4822 | 0.702 | 0.2455 | No |
| 22 | 322614 | arg2 |  |  | 5009 | 0.680 | 0.2470 | No |
| 23 | 100001620 | LOC100001620 |  |  | 6052 | 0.582 | 0.2205 | No |
| 24 | 799963 | gpt2 |  |  | 6058 | 0.581 | 0.2266 | No |
| 25 | 431723 | tha1 |  |  | 7437 | 0.458 | 0.1882 | No |
| 26 | 140620 | prps1a |  |  | 8296 | 0.413 | 0.1657 | No |
| 27 | 406330 | got1 |  |  | 8454 | 0.406 | 0.1652 | No |
| 28 | 323107 | pgam1a |  |  | 8482 | 0.404 | 0.1687 | No |
| 29 | 100006589 | idh1 |  |  | 8531 | 0.401 | 0.1715 | No |
| 30 | 415247\_1 | idh3b |  |  | 8869 | 0.377 | 0.1650 | No |
| 31 | 558487 | eno4 |  |  | 8873 | 0.376 | 0.1690 | No |
| 32 | 678634\_1 | mat2al |  |  | 9250 | 0.350 | 0.1609 | No |
| 33 | 378847 | mtr |  |  | 9320 | 0.346 | 0.1625 | No |
| 34 | 336473\_1 | glulb |  |  | 9542 | 0.330 | 0.1591 | No |
| 35 | 335817 | pkma |  |  | 9917 | 0.309 | 0.1507 | No |
| 36 | 378713 | tktb |  |  | 10030 | 0.301 | 0.1504 | No |
| 37 | 403086 | gpt2l |  |  | 11238 | 0.231 | 0.1149 | No |
| 38 | 794667 | otc |  |  | 11618 | 0.210 | 0.1053 | No |
| 39 | 100149838 | pycr1b |  |  | 11739 | 0.204 | 0.1037 | No |
| 40 | 323329 | mat2aa |  |  | 11774 | 0.202 | 0.1048 | No |
| 41 | 192309 | tpi1a |  |  | 12926 | 0.144 | 0.0702 | No |
| 42 | 324556 | taldo1 |  |  | 13048 | 0.137 | 0.0679 | No |
| 43 | 791730 | got1l1 |  |  | 13108 | 0.135 | 0.0675 | No |
| 44 | 337412 | bcat1 |  |  | 13510 | 0.115 | 0.0561 | No |
| 45 | 317743 | gapdh |  |  | 13695 | 0.107 | 0.0515 | No |
| 46 | 436949 | bcat2 |  |  | 13764 | 0.103 | 0.0504 | No |
| 47 | 406330\_1 | got1 |  |  | 14060 | 0.089 | 0.0421 | No |
| 48 | 557186 | aldh18a1 |  |  | 14170 | 0.084 | 0.0396 | No |
| 49 | 378962 | pah |  |  | 15203 | 0.037 | 0.0075 | No |
| 50 | 322339 | cs |  |  | 15243 | 0.035 | 0.0067 | No |
| 51 | 334116\_1 | eno1a |  |  | 16552 | -0.019 | -0.0343 | No |
| 52 | 334116 | eno1a |  |  | 16760 | -0.025 | -0.0405 | No |
| 53 | 393970 | acy1 |  |  | 16923 | -0.032 | -0.0453 | No |
| 54 | 570368\_1 | arg1 |  |  | 17347 | -0.048 | -0.0581 | No |
| 55 | 100144628 | shmt2 |  |  | 17906 | -0.072 | -0.0748 | No |
| 56 | 447864 | ass1 |  |  | 17996 | -0.075 | -0.0768 | No |
| 57 | 335974 | got2b |  |  | 18318 | -0.090 | -0.0860 | No |
| 58 | 334897\_1 | rpe |  |  | 18449 | -0.096 | -0.0890 | No |
| 59 | 553283 | pcxa |  |  | 18484 | -0.098 | -0.0890 | No |
| 60 | 541347 | mat2b |  |  | 18514 | -0.099 | -0.0889 | No |
| 61 | 100000775 | glula |  |  | 19256 | -0.133 | -0.1107 | No |
| 62 | 100006589\_2 | idh1 |  |  | 19353 | -0.138 | -0.1123 | No |
| 63 | 550259 | pfklb |  |  | 19460 | -0.142 | -0.1141 | No |
| 64 | 406496 | aldoab |  |  | 19616 | -0.149 | -0.1173 | No |
| 65 | 321928 | phgdh |  |  | 19781 | -0.156 | -0.1208 | No |
| 66 | 792692 | aldoca |  |  | 19868 | -0.160 | -0.1218 | No |
| 67 | 606663 | psph |  |  | 20450 | -0.185 | -0.1381 | No |
| 68 | 266987 | cbsb |  |  | 20738 | -0.200 | -0.1449 | No |
| 69 | 369193 | aldocb |  |  | 21049 | -0.218 | -0.1523 | No |
| 70 | 394021 | shmt1 |  |  | 21319 | -0.232 | -0.1583 | No |
| 71 | 566165 | glulc |  |  | 21829 | -0.258 | -0.1715 | No |
| 72 | 327165 | pgam1b |  |  | 22471 | -0.294 | -0.1885 | No |
| 73 | 378963 | eno3 |  |  | 23157 | -0.338 | -0.2064 | No |
| 74 | 402874 | eno2 |  |  | 23379 | -0.353 | -0.2095 | No |
| 75 | 386951 | idh2 |  |  | 23851 | -0.383 | -0.2202 | No |
| 76 | 322055 | cth |  |  | 23997 | -0.391 | -0.2205 | No |
| 77 | 336425 | aldoaa |  |  | 24256 | -0.411 | -0.2242 | No |
| 78 | 393423 | asl |  |  | 25054 | -0.451 | -0.2444 | No |
| 79 | 557716 | nags |  |  | 25514 | -0.486 | -0.2536 | No |
| 80 | 393926 | idh3a |  |  | 25570 | -0.491 | -0.2500 | No |
| 81 | 570106 | pfkla |  |  | 26195 | -0.547 | -0.2637 | No |
| 82 | 568448 | aco1 |  |  | 26535 | -0.582 | -0.2681 | No |
| 83 | 557716\_1 | nags |  |  | 26881 | -0.617 | -0.2723 | No |
| 84 | 100331665 | LOC100331665 |  |  | 27187 | -0.644 | -0.2749 | No |
| 85 | 101886429 | LOC101886429 |  |  | 27507 | -0.674 | -0.2776 | Yes |
| 86 | 415247 | idh3b |  |  | 27612 | -0.688 | -0.2735 | Yes |
| 87 | 572733 | pgam2 |  |  | 27679 | -0.698 | -0.2680 | Yes |
| 88 | 321664 | aldob |  |  | 27744 | -0.709 | -0.2623 | Yes |
| 89 | 100148522 | si:ch211-217a12.1 |  |  | 27762 | -0.711 | -0.2552 | Yes |
| 90 | 678634 | mat2al |  |  | 27805 | -0.716 | -0.2487 | Yes |
| 91 | 557518 | tkta |  |  | 28233 | -0.776 | -0.2538 | Yes |
| 92 | 327512 | psat1 |  |  | 28400 | -0.803 | -0.2503 | Yes |
| 93 | 447836 | pfkma |  |  | 28916 | -0.902 | -0.2568 | Yes |
| 94 | 555623 | cps1 |  |  | 28996 | -0.919 | -0.2493 | Yes |
| 95 | 393668 | eno1b |  |  | 29713 | -1.133 | -0.2596 | Yes |
| 96 | 445094 | pkmb |  |  | 29848 | -1.183 | -0.2510 | Yes |
| 97 | 560827 | prps1b |  |  | 29931 | -1.217 | -0.2404 | Yes |
| 98 | 334195 | mat1a |  |  | 29966 | -1.230 | -0.2282 | Yes |
| 99 | 394138 | asns |  |  | 30083 | -1.278 | -0.2180 | Yes |
| 100 | 792692\_1 | aldoca |  |  | 30223 | -1.351 | -0.2077 | Yes |
| 101 | 568001\_1 | pfkmb |  |  | 30537 | -1.531 | -0.2010 | Yes |
| 102 | 406688 | got2a |  |  | 30634 | -1.617 | -0.1866 | Yes |
| 103 | 407673 | rpia |  |  | 31144 | -2.139 | -0.1794 | Yes |
| 104 | 560753 | tpi1b |  |  | 31226 | -2.286 | -0.1572 | Yes |
| 105 | 561416 | pfkpb |  |  | 31341 | -2.509 | -0.1337 | Yes |
| 106 | 550524\_1 | cbsa |  |  | 31356 | -2.544 | -0.1066 | Yes |
| 107 | 322670\_1 | aco2 |  |  | 31502 | -2.927 | -0.0795 | Yes |
| 108 | 568001\_2 | pfkmb |  |  | 31666 | -3.543 | -0.0463 | Yes |
| 109 | 550579 | idh3g |  |  | 31862 | -4.927 | 0.0009 | Yes |
Table: GSEA details [plain text format]

  

Fig 2: BIOSYNTHESIS\_OF\_AMINO\_ACIDS(DRE01230)      
 Blue-Pink O' Gram in the Space of the Analyzed GeneSet

  

Fig 3: BIOSYNTHESIS\_OF\_AMINO\_ACIDS(DRE01230): Random ES distribution      
 Gene set null distribution of ES for **BIOSYNTHESIS\_OF\_AMINO\_ACIDS(DRE01230)**
